# LncRNA TUG1 functions as a ceRNA for miR‐1‐3p to promote cell proliferation in hepatic carcinogenesis

**DOI:** 10.1002/jcla.24415

**Published:** 2022-04-14

**Authors:** Keke Tang, Di Lv, Lingling Miao, Yushan Mao, Xiaoyan Yu

**Affiliations:** ^1^ The Affiliated Hospital of the Medical School of Ningbo University Ningbo China

**Keywords:** cell apoptosis, cell proliferation, IGF1, LncRNA TUG1, miR‐1‐3p

## Abstract

**Background:**

Hepatocellular carcinoma (HCC) is characterised by high malignancy, metastasis and recurrence, but the specific mechanism that drives these outcomes is unclear. Recent studies have shown that long noncoding RNAs (lncRNAs) can regulate the proliferation and apoptosis of hepatic cells.

**Methods:**

We searched for lncRNAs and microRNAs (miRNAs), which can regulate IGF1 expression, through a bioinformatics website, and predicted that lncRNA taurine‐upregulated gene 1 (TUG1) would have multiple targets for miR‐1‐3p binding, meaning that lncRNA TUG1 played an adsorption role. A double luciferase assay was used to verify the targeting relationship between lncRNA TUG1 and miR‐1‐3p. Western blotting and qPCR were used to verify the targeting relationship between miR‐1‐3p and IGF1, and qPCR was used to verify the regulatory relationship between the lncRNA TUG1‐miR‐1‐3p‐IGF1 axis. CCK‐8 was used to detect the growth activity of miRNA‐transfected L‐O2 cells, and flow cytometry was used to detect cell cycle changes and apoptosis.

**Result:**

The proliferation cycle of L‐O2 cells transfected with miR‐1‐3p mimics was significantly slowed. Flow cytometry showed that the proliferation of L‐O2 cells was slowed, and the apoptosis rate was increased. In contrast, when L‐O2 cells were transfected with miR‐1‐3p inhibitor, the expression of IGF1 was significantly upregulated, and the cell proliferation cycle was significantly accelerated. Flow cytometry showed that the cell proliferation rate was accelerated, and the apoptosis rate was reduced.

**Conclusion:**

LncRNA TUG1 can adsorb miR‐1‐3p as a competitive endogenous RNA (ceRNA) to promote the expression of IGF1 and promote cell proliferation in hepatic carcinogenesis.

## Graphical abstract

Biological function of lncRNA TUG1 in hepatoma cells. A, The growth activity of L‐O2 cells was detected by CCK‐8 after miRNA transfection. B, The proliferation of L‐O2 cells transfected with miRNA was detected by flow cytometry. C, The apoptosis rate of L‐O2 cells after transfection was determined by flow cytometry.

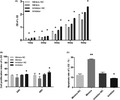

## 1 INTRODUCTION

Liver cancer is one of the most common malignant tumours in the world, ranking second in cancer‐related deaths.^1^ More than 50% of new cases of and deaths due to liver cancer in the world occur in China every year.^2^ Although the supervision and follow‐up of patients with chronic liver diseases have been greatly improved in China in recent years, 70–80% of patients with liver cancer are in an advanced stage of the disease when diagnosed, losing the opportunity for radical surgery, and can only receive palliative treatments.^3^ The 5‐year survival rate of those with liver cancer is no more than 25%, and the prognosis is very poor. These outcomes are related to the characteristics of high malignancy, recurrence and metastasis of liver cancer,^4^, ^5^ so the initial mechanisms of liver cancer are worth exploring. Liver cancer is a polygenic disease. Different genes are involved in different signalling pathways in the development of liver cancer.^6^ Therefore, identifying the key targets for targeted treatment has become a new strategy for cancer prevention and treatment.

Noncoding RNA (ncRNA) refers to RNA that is not translated into polypeptides. Small ncRNAs (sncRNAs) are shorter than 200 nt, and long ncRNAs (lncRNAs) are longer than 200 nt. Although these ncRNAs are not translated into proteins, they can regulate protein expression. LncRNAs can also affect gene expression^7^ through chromosome modification, transcriptional regulation and post‐transcriptional regulation. Taurine‐regulated gene 1 (TUG1) is a lncRNA expressed mainly in the retina and brain and is very important for the normal development of photoreceptors in the retina.^8^ Recent studies have found that lncRNA TUG1 is upregulated in a variety of cancers, including B‐cell malignancy, oesophageal squamous cell carcinoma, ovarian cancer, liver cancer and osteosarcoma.^9^, ^10^, ^11^, ^12^ In these malignant tumours, knockout of TUG1 can inhibit tumour cell proliferation, migration and clone formation. However, the specific mechanism by which lncRNA TUG1 affects cell proliferation and apoptosis remains unknown.

Insulin‐like growth factor 1 (IGF1) is an important growth‐stimulating factor that is mainly synthesised and secreted by hepatocytes. IGF1 regulates cell proliferation by activating the downstream cell signal regulatory protein serine/threonine kinase Akt, and its main receptor IGF1‐R is also increased in liver cancer.^13^ Bioinformatics studies have found that microRNA‐1 (miRNA‐1) can target and regulate the expression of IGF1. MiR‐1‐3p has a sequence antisense to IGF1 mRNA and can silence the expression of the *IGF1* gene through interaction with IGF1 mRNA. LncRNA TUG1 may promote IGF1 expression by inhibiting the silencing effect of miR‐1‐3p on IGF1 expression. Therefore, we aimed to explore the relationship between lncRNA TUG1, miR‐1‐3p and IGF1 and their influence mechanism on the proliferation and apoptosis of liver cells to provide new insights into the prevention and treatment of liver cancer.

## 2 MATERIALS AND METHODS

### 2.1 Prediction of downstream targets of lncRNA TUG1

The interaction between RNA molecules is mediated by miRNA and is based on base complementary pairing. Therefore, the target downstream of TUG1 can be predicted according to relevant software. MiRcode and lncRNABase are bioscience software programmes used to predict the interaction between lncRNAs and miRNAs. Through the above bioinformatics software, we found that there are multiple seed regions of miR‐1‐3p in the TUG1 sequence, and *IGF1* is an important downstream target gene of miR‐1‐3p.

### 2.2 Cell culture

The human hepatic cell line L‐O2 was obtained from Shanghai Institute of Biochemistry and Cell Biology, Chinese Academy of Sciences. The cell lines were cultured in DMEM (Lonza, Walkersville, MD, USA) with 10% foetal bovine serum.

### 2.3 Cell transfection

Hepatic cells were transfected with hsa‐miR‐1‐3p mimics (20 µM), hsa‐miR‐1‐3p inhibitor (20 µM), negative control (NC) inhibitor, or NC mimics using Lipofectamine 2000 reagent (Invitrogen, California, USA) and Opti‐MEM I reduced serum medium (Life Technologies, California, USA) in accordance with the manufacturer's instructions.

### 2.4 Luciferase assay

For the luciferase assay, 293T cells were seeded at 50,000 cells per well in a 24‐well plate. After 24 h, the cells in each well were transfected with a mixture of 0.5 µg of luciferase constructs and 0.5 µl of miR‐1‐3p precursor mimics or control mimics plasmid. At 48 h after transfection, the cells were lysed, and the luciferase activity was measured on a luminometer using a dual‐luciferase reporter assay system (Promega, Shanghai, China) according to the manufacturer's instructions. The luciferase activity was normalised to firefly luciferase activity. The experiments were performed in triplicate and independently repeated at least three times.

### 2.5 Western blot assay

Total protein was extracted from lysed cells. SDS‐PAGE (10%) was used to fractionate 20 µg of the protein samples, which were subsequently transferred to polyvinylidene fluoride membranes. The membranes were blocked with 5% skim milk in TBST for 1 h and incubated with primary antibodies overnight at 4°C. After washing, the membranes were incubated with the secondary antibodies for 1 h at room temperature. Protein signalling was detected using an enhanced chemiluminescence detection system (Tanon, Shanghai, China). The primary antibody was diluted with the newly prepared blocking solution. The dilution ratios of β‐actin (Solarbio, Beijing, China) and IGF1 (Abcam, Shanghai, China) antibodies were 1:5000 and 1:1000, respectively. The secondary antibodies corresponded to each protein primary antibody (mouse secondary antibody 1:3000, rabbit secondary antibody 1:3000).

### 2.6 Quantitative real‐time PCR assay

Total RNA was extracted from transfected target cells by TRIzol, and the concentration of extracted RNA was determined by UV analysis. RNA was reverse transcribed into cDNA. To determine the best primer annealing temperature and template amount, an RT‐PCR pre‐experiment was carried out; the PCR mix was prepared and centrifuged based on an Axygen kit. The above samples were placed into an IQ5 fluorescence quantitative PCR instrument and the SYBR green fluorescence quantitative PCR method was used to analyse the expression of each gene. The design of the miRNA reverse transcription primers and qPCR primers is shown in Table 1.

**TABLE 1:** Primer sequences for miRNA reverse transcription and qPCR

Gene	Primer sequence (5′−3′)
h‐U6 stem‐loop primer	GTCGTATCCAGTGCAGGGTCCGAGGTATTCGCACTGGATACGACAAAATA
hsa‐miR−1‐3p RT primer	GTCGTATCCAGTGCAGGGTCCGAGGTATTCGCACTGGATACGACATACAT
h‐U6 Forward	AGAGAAGATTAGCATGGCCCCTG
h‐U6 Reverse	AGTGCAGGGTCCGAGGTATT
hsa‐miR−1‐3p Forward	GCGCTGGAATGTAAAGAAGT
U primer Reverse	GTGCAGGGTCCGAGGT
IGF1 Forward	TTCACATCTCTTCTACCTG
IGF1 Reverse	TAGCCTGTGGGCTTGTTG
ACTB – Forward	GGCACTCTTCCAGCCTTCC
ACTB – Reverse	GAGCCGCCGATCCACAC
lncTUG1 – Forward	GACTGTTGACCTTGCTGTGAGA
lncTUG1 – Reverse	TGATATGTTGTGGTGTATGTGGG

### 2.7 CCK‐8 assay

Endothelial progenitor cells (EPCs) were seeded into 96‐well plates at a density of 1 × 10^3^ cells/well, and 100 μl of endothelial growth medium (EGM‐2) was added to each well. At 24, 48, 72, 96 and 120 h after inoculation, 10 μl of CCK‐8 reagent was added to each well, and the plate was incubated at 37°C for 2 h. The absorbance at 450 nm (630 nm as a reference) was measured using a microplate reader.

### 2.8 Flow cytometry

The transfected L‐O2 cells in each group were collected, fixed with 70% ethanol, centrifuged at 1000 r/min to remove ethanol and rinsed once with PBS. 300–500 µl PI solution containing 10 µg/ml RNase was then added to resuspend the cells. After incubation in the dark for 30 min, the cell cycle and apoptosis were detected by a BD FACSAria cell sorter (Beckton Dickinson, San Jose, CA, USA).

### 2.9 Statistical analysis

The SPSS 19.0 software (SPSS, Chicago, IL, USA) was used for statistical analysis. All values are presented as the mean ± standard deviation (SD) of three or more independent replicates.^9^ The standard t‐test was performed to compare the differences. *p < 0.05 and **p < 0.01 indicate significant differences.

## 3 RESULTS

### 3.1 *IGF1* is an important downstream target gene of lncRNA TUG1

The downstream targets of lncRNA TUG1 were predicted by using miRcode and lncRNABase. The results showed that there were multiple seed regions of miR‐1‐3p in the TUG1 sequence and that *IGF1* was an important downstream target gene of miR‐1‐3p. Therefore, we constructed the lncRNA TUG1‐miR‐1‐3p‐IGF1 regulatory network.

### 3.2 Luciferase reporter assays showed the binding of lncRNA TUG1 with miR‐1‐3p

**FIGURE 1:**
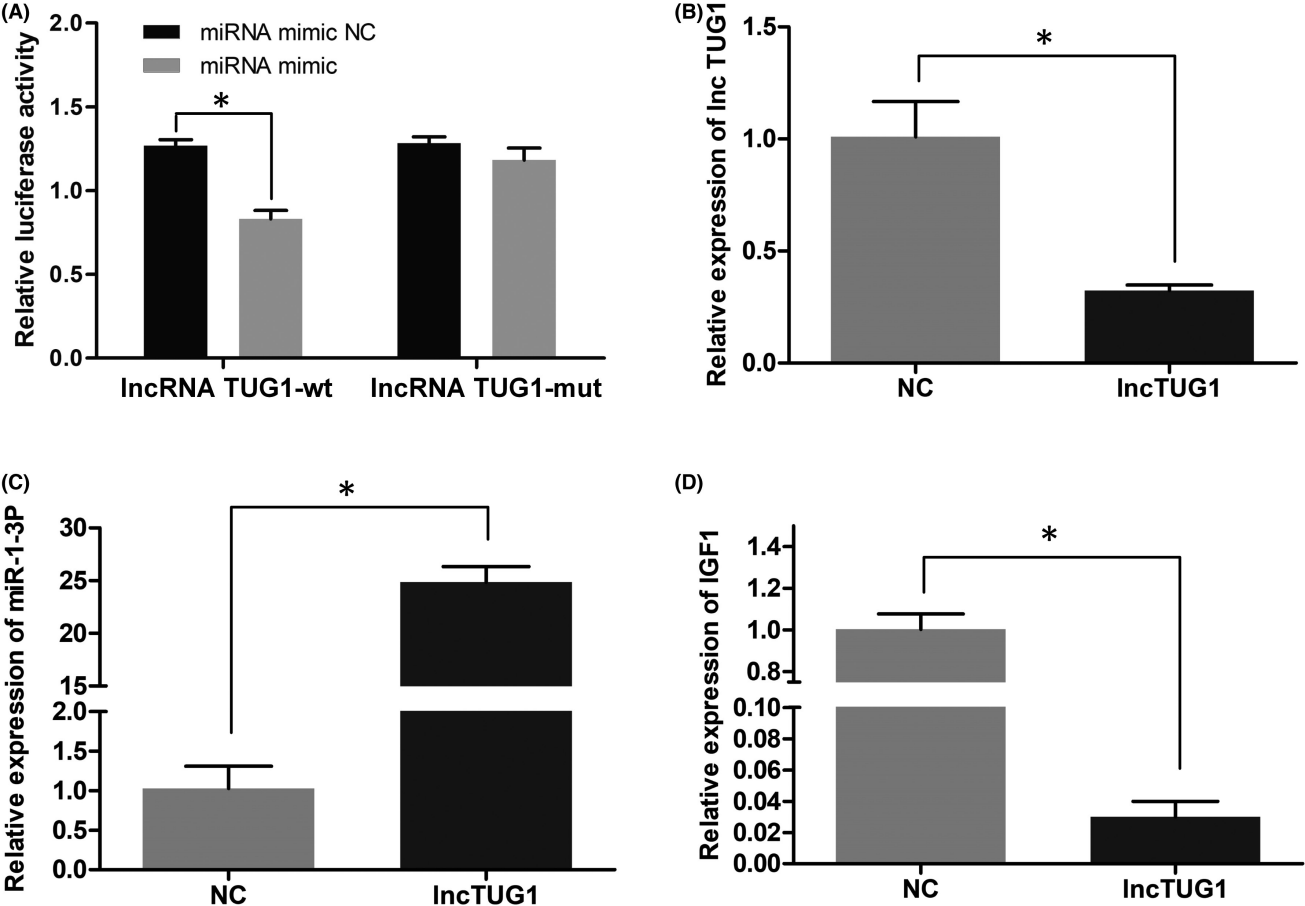
LncRNA TUG1 regulates the expression of miR‐1‐3p and IGF1. (A) miR‐1‐3p mimics significantly reduced the luciferase activity of the wild‐type lncRNA TUG1 gene. (B) The expression of the lncTUG1 gene was detected by qPCR after transient transfection of lncTUG1 siRNA into L‐O2 cells. (C) The expression of the miR‐1‐3p gene was detected by qPCR after transient transfection of lncTUG1 siRNA into L‐O2 cells. (D) The expression of the IGF1 gene was detected by qPCR after transient transfection of lncTUG1 siRNA into L‐O2 cells. (*p < 0.05, **p < 0.01)

To verify the binding of lncRNA TUG1 to miR‐1‐3p, we constructed 3´‐UTR mutant and wild‐type lncRNA TUG1 luciferase reporter gene vectors. The luciferase reporter gene detection results showed that miR‐1‐3p mimics could significantly reduce the luciferase activity of the wild‐type luciferase reporter gene but could not reduce the luciferase activity of the mutant luciferase reporter gene (Figure 1A).

### 3.3 LncRNA TUG1 regulates the expression of miR‐1‐3p and IGF1

The above results confirmed the targeting relationship between lncRNA TUG1 and miR‐1‐3p. After transient transfection of lncTUG1 siRNA into L‐O2 cells, the gene expression levels of miR‐1‐3p and IGF1 were detected by qPCR. The results showed that in L‐O2 cells, with the downregulation of lncTUG1, the expression of miR‐1‐3p was upregulated and the expression of IGF1 was downregulated (Figure 1B, C and D).

### 3.4 Inhibition of miR‐1‐3p can increase the expression of lncRNA TUG1 and IGF1

**FIGURE 2:**
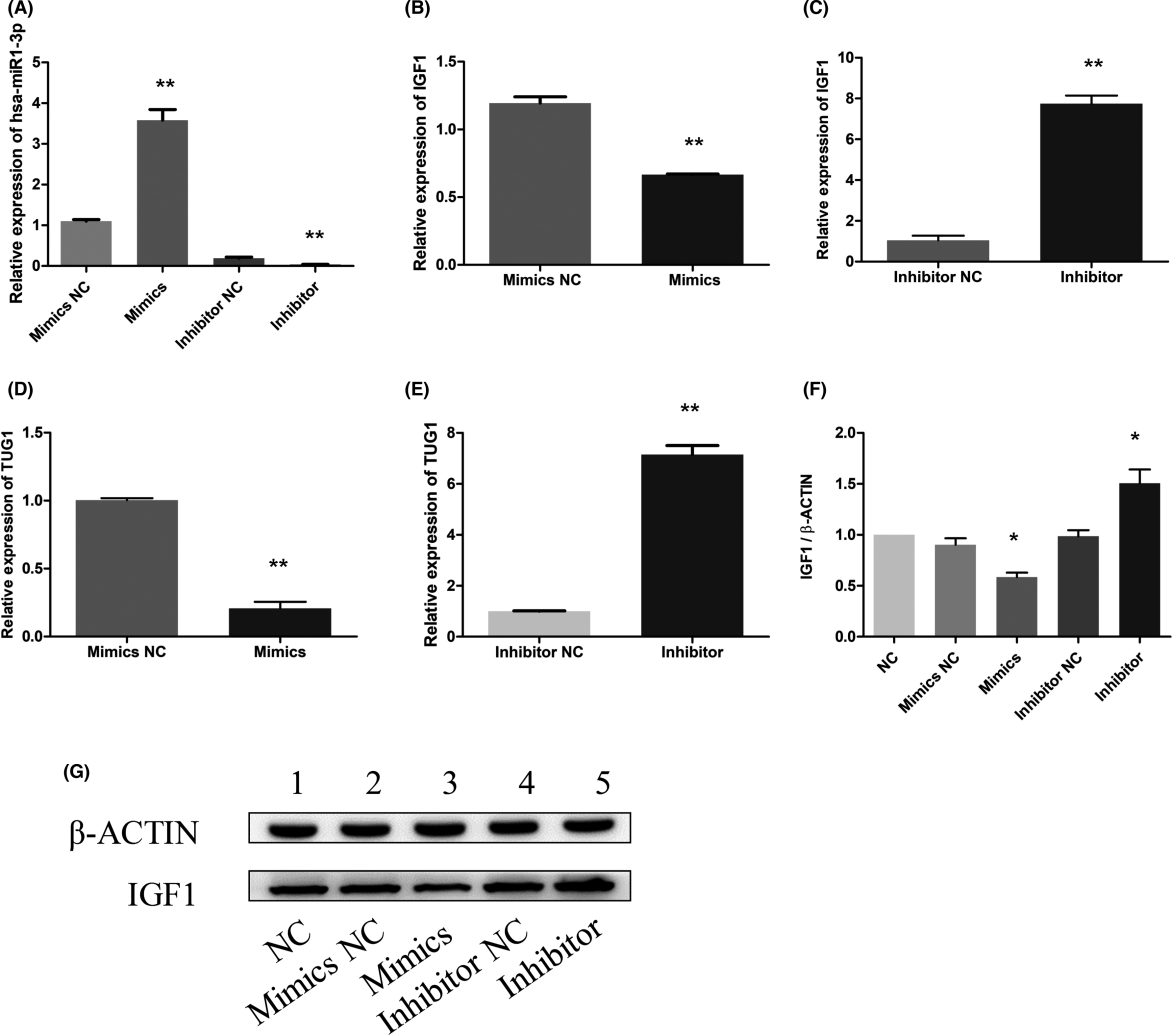
The inhibition of miR‐1‐3p can increase the expression levels of lncRNA TUG1 and IGF1. (A) qPCR was used to detect the expression level of miR‐1‐3p in L‐O2 cells transfected with miR‐1mimics and inhibitor. (B) qPCR was used to detect the expression level of IGF1 after miR‐1mimics transfection. (C) qPCR was used to detect the expression level of IGF1 after inhibitor transfection. (D) qPCR was used to detect the expression level of TUG1 after miR‐1mimics transfection. (E) qPCR was used to detect the expression level of TUG1 after inhibitor transfection. (F) and (G). The expression level of TUG1 was detected by qPCR, and the expression level of IGF1 was detected by WB after transient transfection of L‐O2 cells

From the above results, it can be seen that lncRNA TUG1 can regulate the expression of miR‐1‐3p and IGF1. After transfecting miR‐1‐3p inhibitor and mimics into L‐O2 cells, we found that the expression level of miR‐1‐3p in the mimics group was significantly higher than that in the mimics NC group and that the expression level of miR‐1‐3p in the inhibitor group was significantly lower than that in the inhibitor NC group (Figure 2A). We further compared the expression changes in downstream genes; after miR‐1mimics were transfected into L‐O2 cells, the expression level of IGF1 was significantly downregulated with the increase in miR‐1‐3p, and TUG1 expression was downregulated (Figure 2B,D). After the miR‐1 inhibitor was transfected into L‐O2 cells, compared to that in the inhibitor NC group, the expression level of IGF1 was significantly upregulated in the inhibitor group with the decrease in the miR‐1‐3p level, and TUG1 expression was upregulated (Figure 2C,E).

We further detected the protein expression of miR‐1‐3p inhibitor‐transfected L‐O2 cells with western blotting (WB). The expression level of IGF1 in the inhibitor group was significantly higher than that in the inhibitor NC group, and the expression level of IGF1 in the mimics group was significantly lower than that in the mimics NC group (Figure 2F,G).

### 3.5 Biological function of lncRNA TUG1 in hepatic cells

**FIGURE 3:**
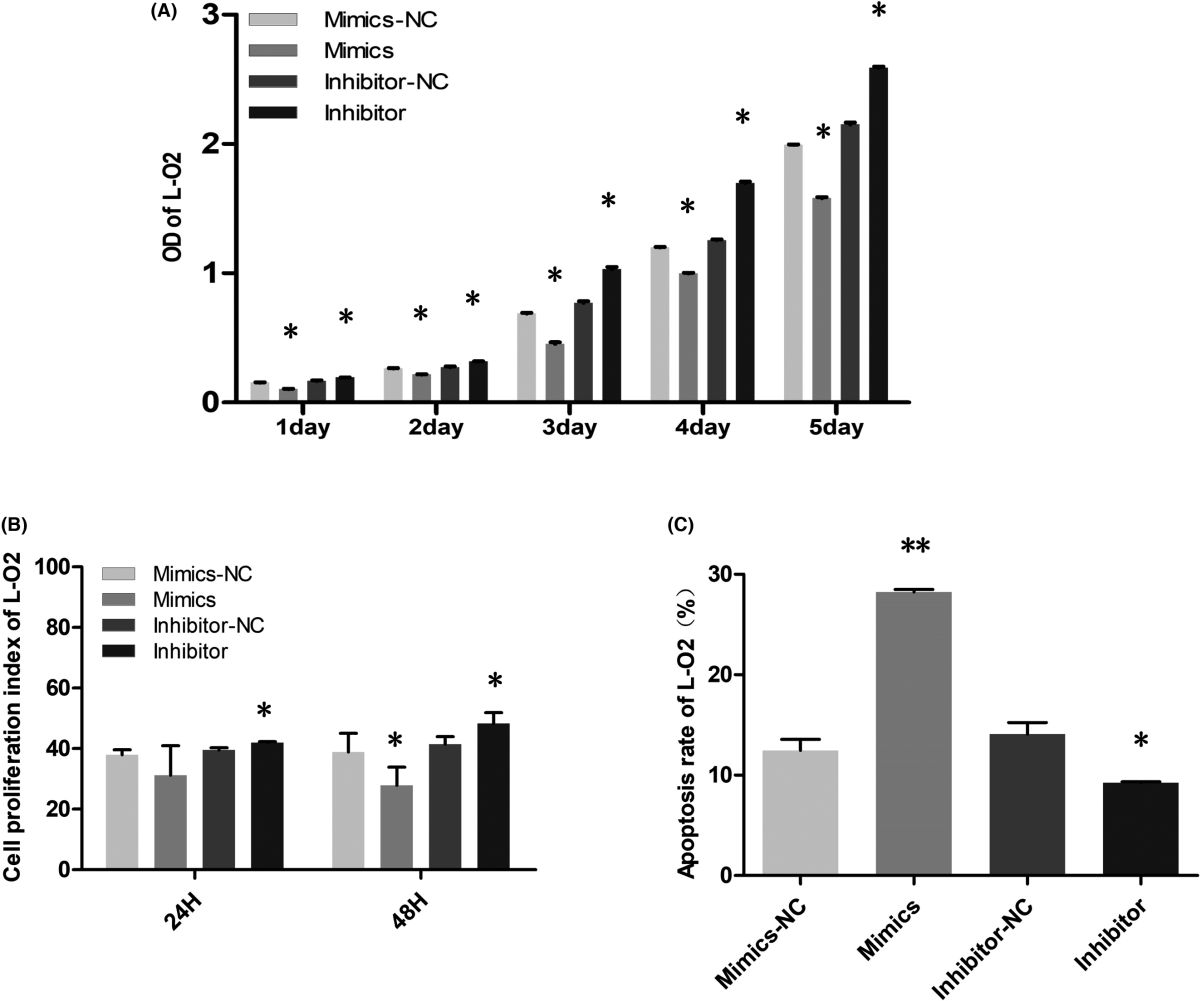
Biological function of lncRNA TUG1 in hepatic cells. (A) The growth activity of L‐O2 cells was detected by CCK‐8 after miRNA transfection. (B) The proliferation of L‐O2 cells transfected with miRNA was detected by flow cytometry. (C) The apoptosis rate of L‐O2 cells after transfection was determined by flow cytometry

**FIGURE 4:**
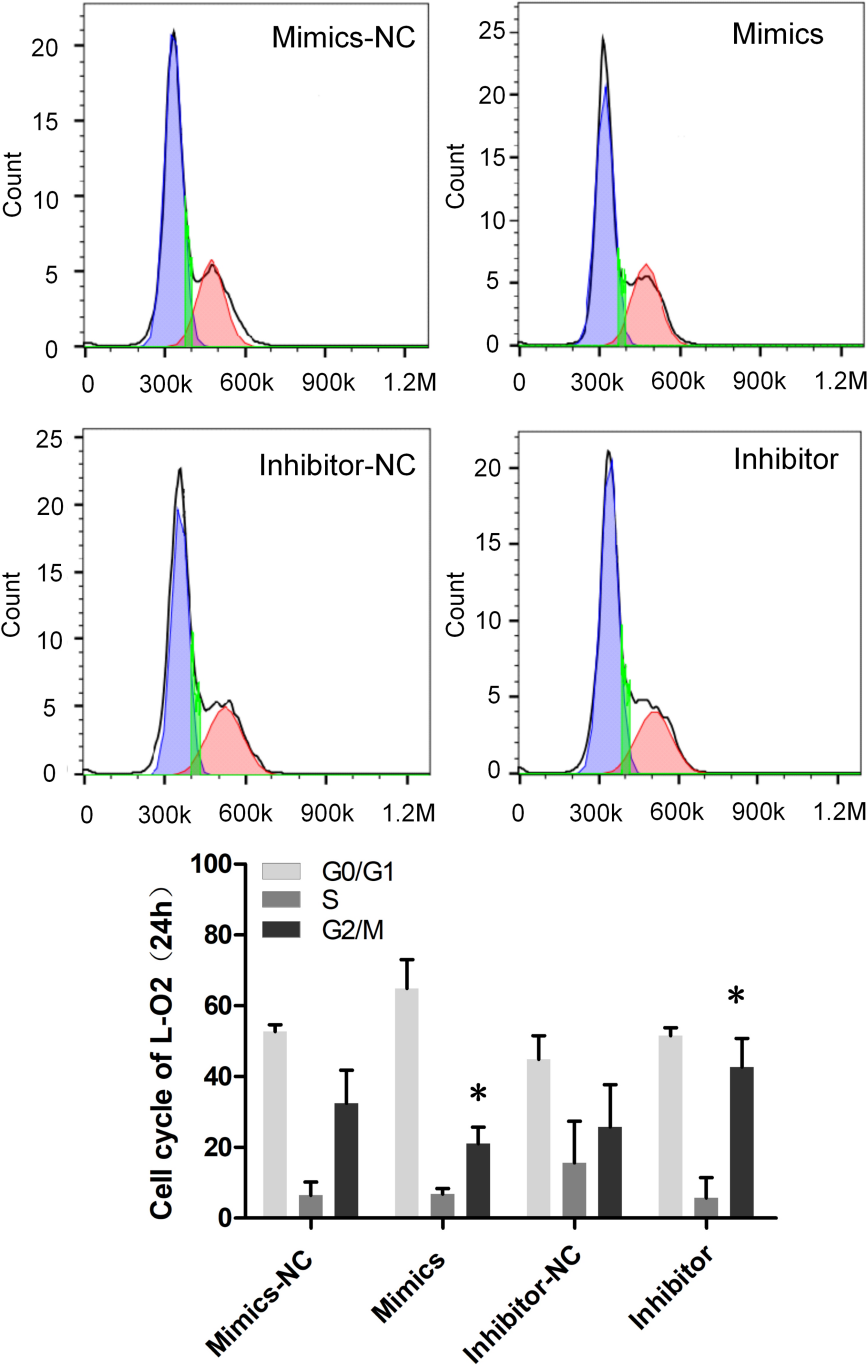
The cell cycle of L‐O2 cells was detected by flow cytometry, after transfection with miRNA for 24 h

**FIGURE 5:**
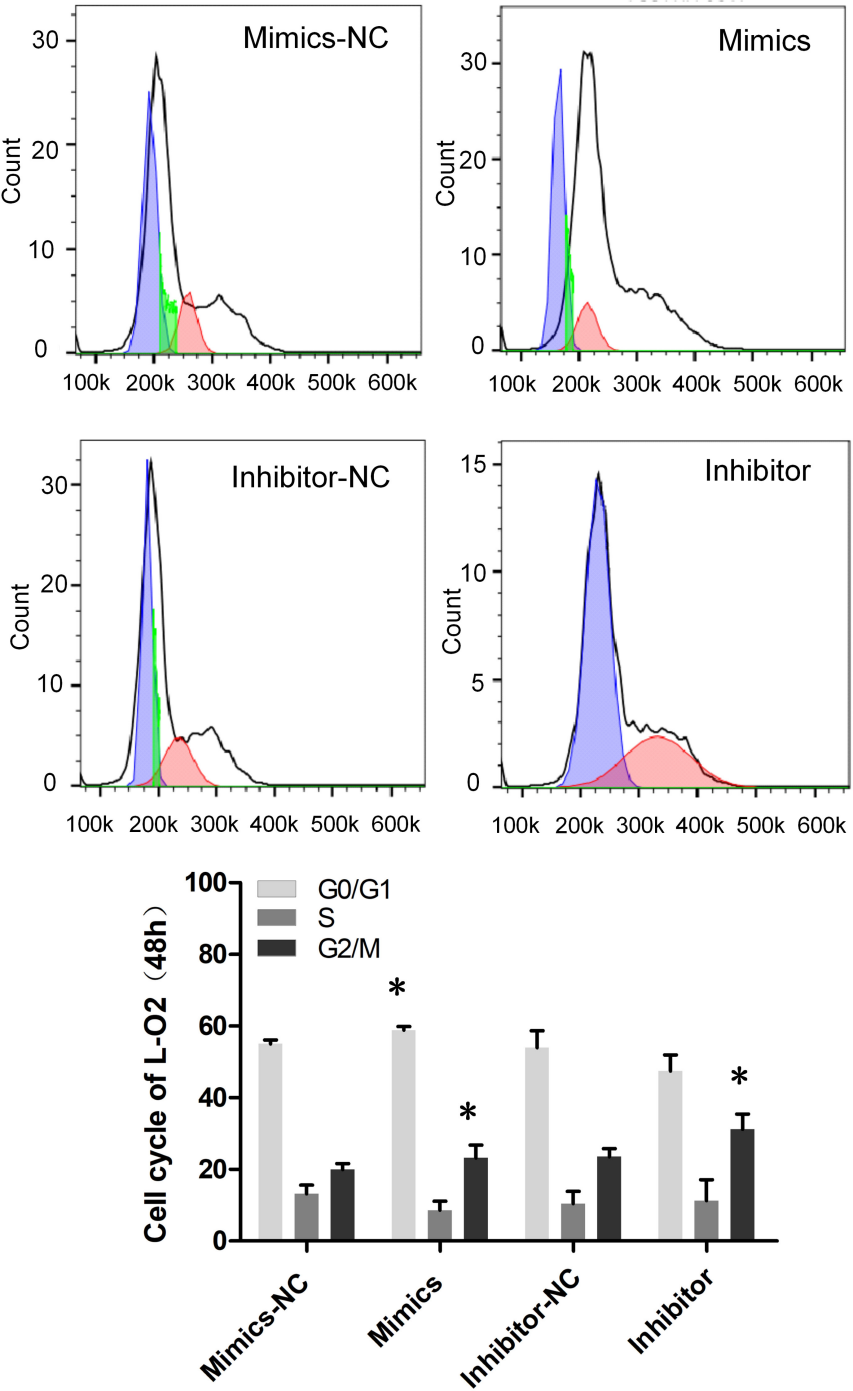
The cell cycle of L‐O2 cells was detected by flow cytometry, after transfection with miRNA for 48 h

Data from Figures 1 and 2 verify the regulatory relationship of the lncRNA TUG1‐miR‐1‐3p‐IGF1 axis. After transfecting miR‐1‐3p mimics and inhibitors into hepatic cells (L‐O2 cells), we detected the cell growth activity with CCK‐8. What stood out in Figure 3A was that the proliferation of hepatic cells in the mimics group was slower than that in the mimics NC group, and the proliferation of hepatic cells in the inhibitor group was higher than that in the inhibitor NC group. We further detected the cell proliferation and apoptosis rates of L‐O2 cells transfected with miRNA by flow cytometry. The results showed that the cell proliferation index of the mimics group was significantly slower than that of the mimics NC group, while that of the inhibitor group was significantly higher than that of the inhibitor NC group (Figure 3B). Compared to that in the mimics NC group, the apoptosis rate in the mimics group tended to increase, while that in the inhibitor NC group tended to decrease (Figure 3C). We further analysed the cell cycle changes in L‐O2 cells at 24 and 48 h after transfection by flow cytometry. The results showed that 24 h after transfection, the proportion of G2/M phase cells in the mimics group was significantly lower than that in the mimics NC group, and the proportion of G2/M phase cells in the inhibitor group was significantly higher than that in the inhibitor NC group (Figure 4); 48 h after transfection, the proportion of G2/M cells in the mimics group was significantly lower than that in the mimics NC group, the proportion of G0/G1 cells was significantly higher than that in the mimics NC group, and the proportion of G2/M cells in the inhibitor group was significantly higher than that in the inhibitor NC group (Figure 5).

## 4 DISCUSSION

LncRNA TUG1 is a lncRNA with 6.7 KB nucleotides. Recent studies have consistently suggested that it can induce the occurrence and development of a variety of tumours, promote endothelial cell proliferation and inhibit apoptosis, but the specific mechanism is still unclear. Many studies have shown that lncRNAs can regulate gene expression at different levels, including chromatin modification, transcriptional regulation and post‐transcriptional regulation. One interesting finding is that LncRNAs can act as ceRNAs to reduce the concentration of miRNAs, resulting in the inhibition of miRNA function in cells.^14^, ^15^ At present, the mechanism of ceRNAs is the most discussed mechanism in lncRNA TUG1. Li J et al.^16^ reported that lncRNA TUG1 adsorbs miR‐132 as a ceRNA to regulate the Hedgehog pathway to promote hepatoma cell proliferation and inhibit apoptosis. In oral squamous cell carcinoma cells, it was found that lncRNA TUG1 promotes cell proliferation and invasion by inhibiting cell mir219 or inducing the expression of FMNL2.^17^ In bladder cancer cells, lncRNA TUG1 increases ZEB2 expression by inhibiting miR‐142 and inhibits the Wnt/β‐catenin pathway to promote cell proliferation and inhibit apoptosis.^18^ For non‐small‐cell lung cancer, lncRNA TUG1 can enhance the sensitivity of cancer cells to chemotherapeutic drugs by interfering with miR‐221‐dependent PTEN inhibition.^19^ In addition, Gao et al.^20^ found that lncRNA TUG1 can also play the role of regulating genes through methylation modification: DMDRMR and IGF2BP3 cooperate to regulate target genes in an m6A dependent manner, thus affecting the prognosis of renal clear cell carcinoma. Chen et al.^21^ confirmed that the mechanism of how EZH2 mediated α‐Actin methylation may depend on TUG1, which promotes the polymerization of cortical F‐actin in synthetic vascular smooth muscle cells. In addition to regulating downstream genes, lncRNA TUG1 can also directly bind protein as a regulator,^22^ thus affecting the development of liver cancer: TUG1 inhibits KLF2 transcription in HCC cells epigenetically by binding to polysulfide protein2 and recruiting it to Kruppel like factor 2 promoter region. TUG1 can also act as a regulator of alpha fetoprotein^23^ and affect the prognosis of HCC. LncRNA TUG1 may participate in the pathological progression of cancer by affecting the activities of cancer cells through different targets. According to bioinformatics analysis, lncRNA TUG1 has multiple targets with a high binding fraction that can bind to miR‐1‐3p, that is, lncRNA TUG1 plays an adsorption role. Therefore, plasmids lncTUG1 gene 3′‐UTR(WT‐1) and lncTUG1 gene 3′‐UTR (mu‐1) were designed to transfect 293T cells. The results showed that for the psiCHECK2‐TUG1‐3'UTR‐WT transfection group, the Rluc/Luc fluorescence ratio of the mimics miR‐1‐3p transfection group was lower than that of the mimics NC group; for the psiCHECK2‐TUG1‐3'UTR‐MU transfection group, there was no significant difference in the Rluc/Luc fluorescence ratio between the mimics miR‐1‐3p transfection group and the mimics NC groups. Therefore, we believe that there is a targeted relationship between the lncRNA TUG1 gene and miR‐1‐3p.

MiR‐1‐3p is a noncoding RNA that plays an important role in the progression of malignant tumours. It inhibits tumour growth and metastasis through different targets. Studies have shown that miR‐1‐3p can suppress the proliferation and invasion of gastric cancer by inhibiting the expression of tin calcium 2,^24^ inhibit the proliferation and metastasis of colorectal cancer cells by regulating YWHAZ‐mediated EMT^25^ and keep down the occurrence of lung adenocarcinoma cells by targeting cytokine 1 protein regulators.^26^ MiR‐1‐3p can also prevent cell proliferation, invasion and migration by regulating the BDNF‐TrkB signalling pathway in bladder cancer^27^ and upregulating SFRP1 expression.^28^ MiR‐1‐3p inhibits the proliferation of hepatocellular carcinoma by targeting Sox9.^29^ Through bioinformatics analysis, it was found that miRNA‐1 can target and regulate the expression of IGF1. MiR‐1‐3p has an antisense sequence with IGF1 mRNA and can silence the expression of the *IGF1* gene through interaction with IGF1 mRNA. Kun L et al validated *IGF1* as a target gene of miR‐1 by luciferase assay.^30^ Therefore, in this study, miR‐1 mimics were transfected into the hepatic cell line L‐02, and the expression of IGF1 in each group was detected by WB. The results showed that the expression of IGF1 in the mimics group was significantly lower than that in the control group, and the expression of IGF1 in the inhibitor group was significantly higher than that in the control group. These results further demonstrated the targeting relationship between miR‐1‐3p and IGF1.

To systematically demonstrate the regulatory effect of the lncRNA TUG1‐miR‐1‐3p‐IGF1 axis, this study further performed lncTUG1 siRNA transient transfection of L‐O2 cells. And qPCR detection was performed on each group of cells. The results showed that with the downregulation of lncTUG1, the expression of miR‐1‐3p and IGF1 in L‐O2 cells was upregulated and downregulated, respectively. After verifying the role of the lncRNA TUG1‐miR‐1‐3p‐IGF1 regulatory axis, CCK‐8 was further used to detect the cell growth state, and flow cytometry was used to detect the cell cycle and apoptosis rate. Compared to those in the mimics NC group, the cell proliferation cycle was significantly slowed down and the apoptosis rate was increased in the miR‐1 mimics group. However, the cell proliferation cycle was significantly accelerated, and the apoptosis rate was decreased in the miR‐1 inhibitor group, compared to those in the inhibitor NC group. Based on the above experimental results, this study demonstrated the regulatory axis of lncRNA TUG1‐miR‐1‐3p‐IGF1, verified its regulatory effect on the proliferation and apoptosis of hepatic cells, and provided a new target for and insight into the prevention and treatment of hepatoma. However, due to funding and time constraints, this study only explored the interaction between lncRNA TUG1, miR‐1‐3p and IGF1 at the cellular level and did not verify this ceRNA mechanism at the animal level. This project will be included in our next research plan.

## CONFLICT OF INTEREST

The authors declare no conflicts.

## Data Availability

The data that support the findings of this study are available from the corresponding author upon reasonable request.
